# Microphtalmie colobomateuse

**DOI:** 10.11604/pamj.2014.17.308.3976

**Published:** 2014-04-22

**Authors:** Zineb Jaja, Salim Belhassan

**Affiliations:** 1 Université Mohammed V Souissi, Service d'Ophtalmologie A de l'Hôpital des Spécialités, Centre Hospitalier Universitaire, Rabat, Maroc

**Keywords:** Microphtalmie, cécité, conseil génétique, microphthalmia, blindness, genetic counseling

## Image en médicine

La microphtalmie colobomateuse est une maladie rare, c'est une cause importante de cécité congénitale, avec une prévalence de 1.9-3.5/10 000 naissances vivantes. Nous rapportons le cas d'une fille de 7 ans présentant une microphtalmie colobomateuse unilatérale isolée. Il s'agit d'une fille âgée de 7 ans sans antécédents personnels ou familiaux présentant depuis sa naissance une microphtalmie unilatérale. Les microphtalmies colobomateuses sont dues à un défaut de fermeture de la fente embryonnaire à la 7e semaine de vie intra-utérine. Les facteurs génétiques et environnementaux peuvent jouer un rôle dans cette malformation de l'oeil. La microphtalmie colobomateuse est une atteinte oculaire rare devant laquelle L'examen clinique ophtalmologique reste à la base du diagnostic précis de la maladie ainsi que l'examen des apparentés, porteurs d’éventuels gènes mutés impliqués dans la maladie, est systématique ainsi que le conseil génétique.

**Figure 1:**
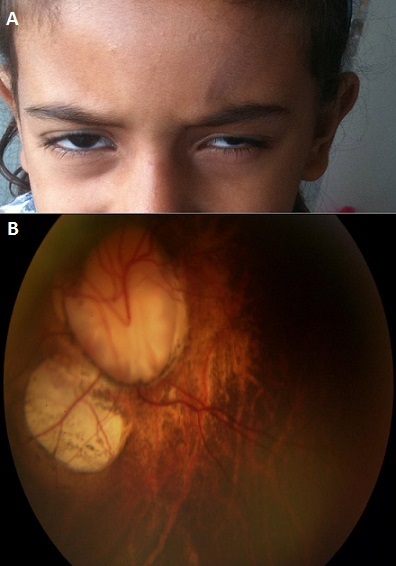
A) microphtalmie microphtalmie de l’œil gauche; B) colobome choriorétinien

